# CNN-Based Classifier as an Offline Trigger for the CREDO Experiment

**DOI:** 10.3390/s21144804

**Published:** 2021-07-14

**Authors:** Marcin Piekarczyk, Olaf Bar, Łukasz Bibrzycki, Michał Niedźwiecki, Krzysztof Rzecki, Sławomir Stuglik, Thomas Andersen, Nikolay M. Budnev, David E. Alvarez-Castillo, Kévin Almeida Cheminant, Dariusz Góra, Alok C. Gupta, Bohdan Hnatyk, Piotr Homola, Robert Kamiński, Marcin Kasztelan, Marek Knap, Péter Kovács, Bartosz Łozowski, Justyna Miszczyk, Alona Mozgova, Vahab Nazari, Maciej Pawlik, Matías Rosas, Oleksandr Sushchov, Katarzyna Smelcerz, Karel Smolek, Jarosław Stasielak, Tadeusz Wibig, Krzysztof W. Woźniak, Jilberto Zamora-Saa

**Affiliations:** 1 Institute of Computer Science, Pedagogical University of Krakow, 30-084 Kraków, Poland; olaf.bar@up.krakow.pl (O.B.); lukasz.bibrzycki@up.krakow.pl (Ł.B.); 2 Faculty of Computer Science and Telecommunications, Cracow University of Technology, 31-155 Kraków, Poland; nkg@pk.edu.pl (M.N.); katarzyna.smelcerz@pk.edu.pl (K.S.); 3 Department of Biocybernetics and Biomedical Engineering, AGH University of Science and Technology, 30-059 Kraków, Poland; krz@agh.edu.pl (K.R.); m.pawlik@cyfronet.pl (M.P.); 4 Institute of Nuclear Physics, Polish Academy of Sciences, 31-342 Kraków, Poland; slawomir.stuglik@ifj.edu.pl (S.S.); david.alvarez@ifj.edu.pl (D.E.A.-C.); kevin.almeida-cheminant@ifj.edu.pl (K.A.C.); Dariusz.Gora@ifj.edu.pl (D.G.); Piotr.Homola@ifj.edu.pl (P.H.); Robert.Kaminski@ifj.edu.pl (R.K.); Justyna.Miszczyk@ifj.edu.pl (J.M.); oleksandr.sushchov@ifj.edu.pl (O.S.); jaroslaw.stasielak@ifj.edu.pl (J.S.); Krzysztof.Wozniak@ifj.edu.pl (K.W.W.); 5 NSCIR, Thornbury, ON N0H2P0, Canada; tandersen@nscir.ca; 6 Applied Physics Institute, Irkutsk State University, 664003 Irkutsk, Russia; nbudnev@api.isu.ru; 7 Bogoliubov Laboratory of Theoretical Physics, JINR, 6 Joliot-Curie St, 141980 Dubna, Russia; 8 Aryabhatta Research Institute of Observational Sciences (ARIES), Manora Peak, Nainital 263001, India; alok@aries.res.in; 9 Astronomical Observatory, Taras Shevchenko National University of Kyiv, UA-01033 Kyiv, Ukraine; bohdan_hnatyk@ukr.net (B.H.); alenamozgova@ukr.net (A.M.); 10 Astrophysics Division, National Centre for Nuclear Research, 28 Pułku Strzelców Kaniowskich 69, 90-558 Łódź, Poland; mk@zpk.u.lodz.pl; 11 Astroparticle Physics Amateur, 58-170 Dobromierz, Poland; mpknap@wp.pl; 12 Institute for Particle and Nuclear Physics, Wigner Research Centre for Physics, Konkoly-Thege Miklós út 29-33, 1121 Budapest, Hungary; kovacs.peter@wigner.hu; 13 Faculty of Natural Sciences, University of Silesia in Katowice, Bankowa 9, 40-007 Katowice, Poland; bartosz.lozowski@us.edu.pl; 14 Joint Institute for Nuclear Research, Joliot-Curie Street 6, 141980 Dubna, Russia; vnazari@jinr.ru; 15 Institute of Secondary Education, Highschool No. 65, 12000 Montevideo, Uruguay; mrosas@docente.ceibal.edu.uy; 16 Institute of Experimental and Applied Physics, Czech Technical University in Prague, Husova 240/5, 110 00 Prague, Czech Republic; karel.smolek@utef.cvut.cz; 17 Faculty of Physics and Applied Informatics, University of Lodz, 90-236 Łódź, Poland; t.wibig@gmail.com; 18 Departamento de Ciencias Fisicas, Universidad Andres Bello, Santiago 8370251, Chile; jilberto.zamora@unab.cl

**Keywords:** image sensors, global sensor network, gamification, citizen science, convolutional neural networks, image classification, deep learning, CREDO

## Abstract

Gamification is known to enhance users’ participation in education and research projects that follow the citizen science paradigm. The Cosmic Ray Extremely Distributed Observatory (CREDO) experiment is designed for the large-scale study of various radiation forms that continuously reach the Earth from space, collectively known as cosmic rays. The CREDO Detector app relies on a network of involved users and is now working worldwide across phones and other CMOS sensor-equipped devices. To broaden the user base and activate current users, CREDO extensively uses the gamification solutions like the periodical Particle Hunters Competition. However, the adverse effect of gamification is that the number of artefacts, i.e., signals unrelated to cosmic ray detection or openly related to cheating, substantially increases. To tag the artefacts appearing in the CREDO database we propose the method based on machine learning. The approach involves training the Convolutional Neural Network (CNN) to recognise the morphological difference between signals and artefacts. As a result we obtain the CNN-based trigger which is able to mimic the signal vs. artefact assignments of human annotators as closely as possible. To enhance the method, the input image signal is adaptively thresholded and then transformed using Daubechies wavelets. In this exploratory study, we use wavelet transforms to amplify distinctive image features. As a result, we obtain a very good recognition ratio of almost 99% for both signal and artefacts. The proposed solution allows eliminating the manual supervision of the competition process.

## 1. Introduction

### 1.1. CREDO Project

The Cosmic Ray Extremely Distributed Observatory (CREDO) is a global collaboration dedicated to observing and studying cosmic rays (CR) [1] according to the Citizen Science paradigm. This idea underpinned some other similar particle detection initiatives like CRAYFIS [2,3,4,5,6] and DECO [7,8,9,10]. The CREDO project collects data from various CR detectors scattered worldwide. Note that according to the project’s open access philosophy, the collected data are available to all parties who want to analyse them. Given the large amount of potential hits registered in these experiments and the fact that only a fraction of them are attributable to particles of interest (mostly muons), effective on-line or off-line triggers are a must. The on-line muon trigger described in [4] was based on the CNN with a lazy application of convolutional operators. Such an approach was motivated by the limited computational resources available in mobile devices. Here, we propose an alternative approach to the CNN-based trigger design aimed principally at off-line use. However, the moderate size of convolutional layers in our design in principle allows for use also with the limited resources of smartphones.

CR are high-energy particles (mostly protons and atomic nuclei) which move through the space [11]. They are emitted by the Sun or astrophysical objects like supernovae, supermassive black holes, quasars, etc. [12]. CR collide with atoms in the Earth’s atmosphere thus producing secondary particles that can undergo further collisions, finally resulting in particle air showers that near the Earth surface consist of various particles, mainly photons and electrons/positrons but also muons. Muons are of principle interest to us because their signatures are easily distinguishable from other particles. Moreover, it is guaranteed that they are of cosmic origin as there are no terrestrial sources for muons. Such air showers can be detected by various CR detectors. CR studies provide an alternative to studying high-energy particle collisions in accelerators [13] and in terms of energies available surpass them by several orders of magnitude. As the CR are ionising radiation, they can cause the DNA mutations [14], damage of hardware, data storage or transmission [15]. Monitoring the intensity of the radiation flux (cosmic weather) is also important for manned space missions [16] and humans on Earth [17]. Existing detector systems are operating in isolation, whereas CR detectors used by the CREDO collaboration operate as part of a global network. This may provide new information on extensive air showers, or Cosmic Ray Ensembles [1]. The CREDO project is gathering and integrating detection data provided by users. The project is open to everybody who wants to contribute with their own detector. Most of the collected data comes from smartphones running the CREDO Detector app, operating on the Android system [18]. The physical process behind registering cosmic rays with the app is identical to that used by silicon detectors in high-energy physics experiments. The ionising radiation interacts with the camera sensor and produces electron–hole pairs [19]. Then, the algorithm in the app analyses the image from the camera and searches for CR hits. Signals qualified as hits of cosmic rays are then sent to the CREDO server.

The overall scale of the CREDO observation infrastructure and the data collected so far can be summarised in the following statistics (approximate values as of February 2021): over $1.2\times 10^{4}$ unique users, over $1.5\times 10^{4}$ physical devices, over $1.0\times 10^{7}$ candidate detections registered in a database, and the total operation time of $3.9\times 10^{5}$ days, i.e., more than 1050 years.

### 1.2. Gamification as a Participation Driver

To arouse interest in cosmic ray detection with the CREDO Detector application among primary and secondary school pupils, university students and all interested astroparticle enthusiasts, an element of gamification was introduced. One of these elements is the “Particle Hunters” competition. In this competition, each participant teams up with other participants under the supervision of a team coordinator who is usually a teacher from their educational organisation. Each participating team’s goal is to capture as many good cosmic ray particle candidates as possible using the above-mentioned CREDO Detector application. The competition is played in two categories: League and Marathon.

**Figure 1:**
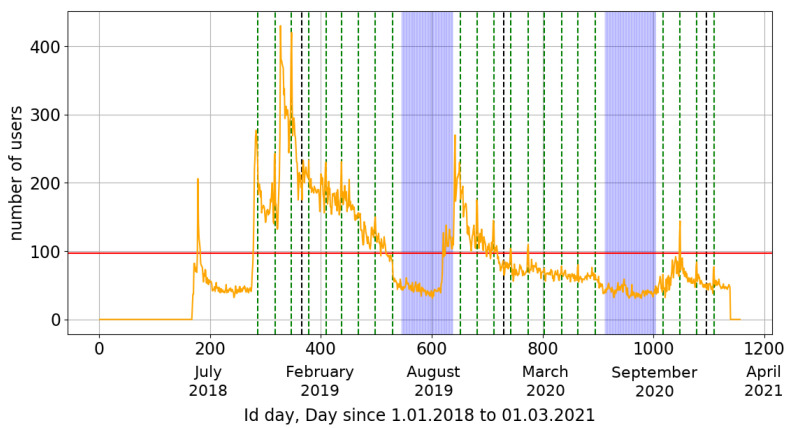
Daily activity of the CREDO Detector application users from the premiere of the application (June 2018) to 1 March 2021. Green lines show the days of the event in the competition for schools. Dashed black lines indicate the beginning and end of a given year. Horizontal red line reflects the average daily number of active users (97). Blue areas indicate areas between two editions of the competition (early July to early October).

- Competition in the League category: Consists of capturing particles during one selected night of the month. During the competition, each month, on the night of the 12th to the 13th of the month, competition participants launch the CREDO Detector application between 9 pm and 7 am (local time of each team). The winner of the competition is chosen based on the number of particles captured during that one night. In Figure 1, the day on which this event occurred (League) is indicated by a dashed vertical green line.
- Competition in the Marathon category: Members of the team participating in this category launch the CREDO Detector application at any time during the competition. At the end of the contest, the total number of detections made by all team members for the entire duration of the event is calculated, including detections made for the League category.

The competition lasts 9 months. The current third edition runs from 21 October 2020 to 18 June 2021. The role of gamification in the CREDO project can be observed in a plot of the daily activity of users of the CREDO Detector Application shown in Figure 1. A significant increase in user activity during each edition of the “Particle Hunters” competition can be observed.

In particular, it can be seen that the daily activity of the CREDO application users has changed since the competition inception. Statistically, about 97 users are active daily, but there is a decrease in the holiday season and an increased activity of users in periods of competitions. There is also a visible decrease during the pandemic—where the possibilities of advertising the application (e.g., at science festivals) are very limited. The horizontal axis shows the number of days from 1 January 2018. The application was launched (start) in June 2018.

The statistics of the last (completed) edition of the competition is shown in Table 1. The table compares results of all users with those participating in the competition.

The above statistics show that during the competitions most of detections, i.e., 67%, come from the competitors. This proves the positive impact of the gamification upon the CREDO project performance. Unfortunately, the data collection process is exposed to the cheating users, trying to deliver as many hits as possible. Significant part of data are thus unusable for the research, because the percentage of good detection candidates decreases from 46% to 30%, see Table 1. Therefore, to be able to fully exploit the potential of gamification, an efficient, fair, and intelligent detection filtering mechanism is required, and this is where machine learning capabilities come into play. More information about the competition can be found on the official “Particle Hunters” website [20].

### 1.3. Data Management

The events being sent to the server can be corrupted. This is because the detection from CCD/CMOS sensors is strongly dependent on the correct use. The Android app provided by CREDO collaboration is working on the so-called dark frame, i.e., the image registered with the camera tightly covered. A user can, however, produce fake detections, by the corruption of the dark frame, e.g., with not fully covered sensor or use of an artificial light sources to simulate cosmic ray hits. More obvious cases related to fakes and hardware malfunction can be relatively easy recognised and filtered. The simplest off-line filter that is used in the competition is the anti-artefact filter, which consists of three parts of detection analysis [19]:

- Coordinate analysis—more than two events at the same location on two consecutive frames are marked as hot pixels because they are statistically incompatible with the muon hit rate.
- Time analysis—hits are rejected if more than 10 detections are registered on a given device per minute, which is also incompatible with the expected muon hit rate.
- Brightness analysis—the frame cannot contain more than 70 pixels with luminance greater than 70 in the greyscale.

Requirements defined above reduce the number of detections from 10.5 million to about 4 million. More specifically, based on the time analysis, about 5.8 million hits were rejected, while 380 thousand hits were rejected based on the brightness analysis, with another 0.8 million rejected based on the coordinate analysis.

## 2. ANN-Based Method to Remove Artefacts

Analysis of data collected from various types of sensors is one of the most important driving forces in the development of computational intelligence methods. Significant challenges are especially issues related to the multiplicity of sources (types of sensors), the operation of sensors in distributed systems, the exponential increase in the volume of information received from them and very often the requirement to carry out the analysis in the real-time regime. Tasks related to classification and recognition are often approached by well-known statistical models [21,22], such as SVM [23], ANN [24] or RF [25]. Recently, deep learning models, based on various variants of neural networks, such as CNN [26], RNN [27] or GNN [28], are also experiencing a renaissance. Depending on the area and specificity of applications, very complex approaches are used, often utilising integration and combination of many techniques, which is particularly visible in case of interdisciplinary problems occurring, for example, in such selected fields as medicine [29,30], education [31,32], metrology [33,34,35], biometrics [36,37], learning of motor activities [38,39] or gesture recognition [40,41].

In image classification and recognition tasks, convolutional architectures (CNNs), regardless of depth, have a structural advantage over other types of statistical classifiers and usually outperform them. Therefore, in this paper, we chose to design an approach based on deep convolutional networks. Several recognised CNN-based classifier models were considered, including AlexNet [42], ResNet-50 [43], Xception [44], DenseNet201 [45], VGG16 [46], NASNetLarge [46] and MobileNetV2 [47]. The possibilities of using the concept of transfer learning were also analysed, where such networks are pretrained for large, standardised data sets, such as ImageNet [48]. The transfer learning approach to classifying the CREDO data was already discussed in [49]. Due to the peculiarity of the problem, quite unusual input data and a small spatial size of the signal in the images (only a few to a maximum of several dozen pixels), we decided to develop a dedicated architecture tailored to the specifics and requirements of the problem. To obtain the optimal classifier we explored different architectures (taking into account a constraint related to the relatively low resolution of input images) and available hyperparameter values, like learning rate, batch size, solver, regularisation parameters, pooling size, etc. Section 2.2 presents the best classifier setup we found.

### 2.1. Experiment Design

**Figure 2:**
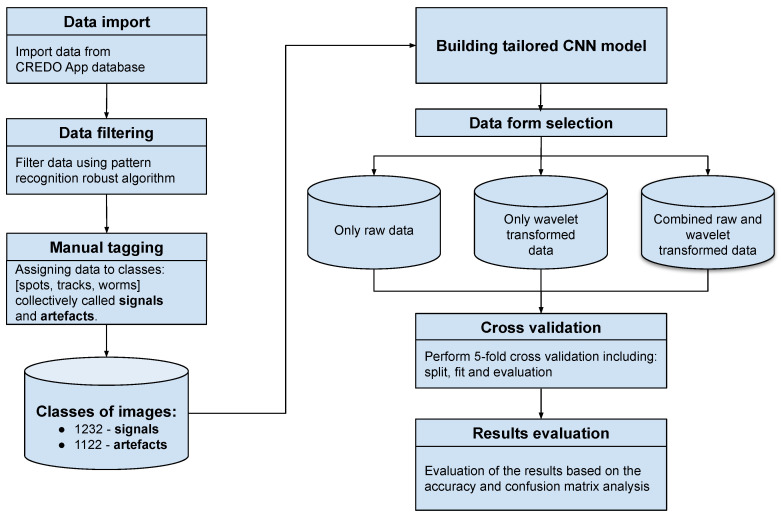
Computation experiment flow chart.

The experiment was performed, and its flow chart is presented in Figure 2. The experiment consists of the following steps, where some of them are described in next subsections in detail:

- Data import. The source data are stored in the CREDO App database [18]. Data used in this experiment were imported and stored in a more flexible format for further computation.
- Data filtering. Due to a huge amount of useless data, including very typical and obvious artefacts, the robust and deterministic filtering algorithm with high specificity was applied [50]. As a result, all non-artefact data are retained.
- Manual tagging. Manual tagging using web-based software [51] by five independent researchers was performed. As a result, four classes of images were obtained: 535 spots, 393 tracks, 304 worms and 1122 artefacts. However, in this study we are focused on a binary classification. Therefore, spots, tracks and worms made up one class (called collectively signal) and the artefacts the other. Given a manually labelled dataset, we can roughly estimate the annotators’ classification uncertainty in terms of the mean and standard deviation of the number of votes cast for an image that is a signal or artefact. Five people did a manual classification. We used samples whose classification was almost unanimous, i.e.,
  - from 1232 signals 815 were classified by 5 of 5 and 417 were classified by 4 of 5,
  - from 1122 artefact are classified by 5 of 5.
  Therefore, an average signal vote can be calculated according the Equation (1):
  (1) $$\frac{\left(5\times 815+4\times 417\right)}{\left(815+417\right)}=4.7$$
  Respective probabilities of a given vote number for signal are
  − 5/5 votes probability: 66%,
  − 4/5 votes probability: 34%,
  − standard deviation of votes: 0.48.
  Thus, the overall vote number probability is 4.7±0.5, which gives 10% of relative uncertainty.
- Building a CNN model. The main part of the experiment is the Artificial Neural Network model based on Convolutional Neural Network described in Section 2.2.
- Data preprocessing. We consider three approaches to preparing the input data: feeding raw data (Section 3.1), feeding wavelet transformed data (Section 3.2) and feeding the fusion of raw and wavelet transformed data (Section 3.3).
- Cross-validation. The model was trained and tested in a non-stratified repeated 5-fold cross-validation standard procedure, thus resulting in 25 classification results.
- Results evaluation. Finally, the results obtained were evaluated using accuracy calculated as the fraction of correct classifications to overall classifications and are presented in Section 3.

The computations were optimised with respect to various single wavelet transformations performed during preprocessing.

### 2.2. CNN Model and Its Architecture

**Figure 3:**
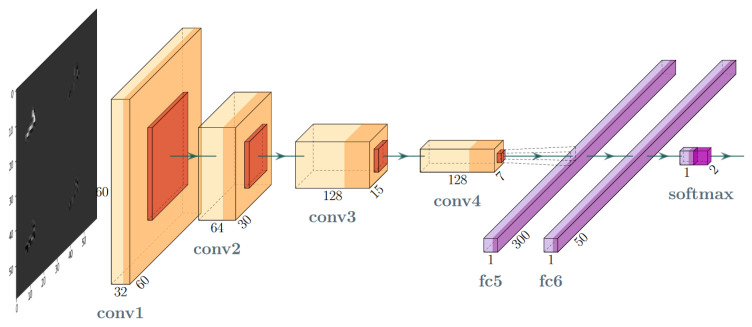
Layer-oriented signal flow in the convolutional network (CNN) artefact filtration model.

The Convolutional Neural Network (CNN) model shown in Figure 3 was build to perform the experiment. The model is moderately deep and its convolutional layers are moderately wide. The motivation for such an architecture was the potential to use it as the lightweight trigger in the online applications. Therefore, the network we used had a typical architecture including convolutional, pooling and fully-connected layers. In this architecture, the model hyperparameters to be configured include the size of filters and kernels, the activation function in convolutional layers, the size of the pool in pooling layers, the output space dimensionality, the activation function and kernel initialiser as well as their regularisers in fully-connected layers.

The best hyperparameter combination was found manually by performing many trial and error cycles. Finally, we used the architecture that consisted of the layers and its parameters which are listed in Table 2. The optimisation algorithm RMSProp [52] using a batch size of 64 was used as the solver. Additionally, for the fully-connected (dense) layers, a combined L1 and L2 regularisation (so-called Elastic Net [53]) with coefficients of 0.01 was applied.

### 2.3. Applying Wavelet Transforms as Feature Carriers

The CNN input layer can be fed with raw images that in case of CREDO data are 60 × 60 (RGB) three-layer colour images. Given the great diversity of artefact images in terms of types and shapes we came up with a design which focuses on general image properties like the shape of the border or the connectedness of the image pattern. These general properties can be amplified by applying wavelet transformation. As a result, one obtains the averaged image along with horizontal and vertical fluctuations which amplify horizontal and vertical border components, respectively. Accordingly, the raw data are subject to preprocessing as per the recipe below. The first preprocessing step is a greyscale conversion, which is implemented by summing up the channels. This step is aimed to remove a redundant information which does not carry any physical interpretation. The colour of the pixel is associated with the colour filter, overlaid on the CMOS array, that happened to be hit during detection. This is basically a random event and is not correlated to radiation species. The next step is the noise reduction. As the analysed images were of different overall brightness, we decided to apply a noise cut-off algorithm which depends on the average brightness. Moreover, images marked as “artefacts” usually differ from “non-artefacts” by a few standard deviations in brightness. The two above mentioned quantities were used to define the cut-off threshold, i.e., average and standard deviation of brightness (Equations (2) and (3)). The threshold was determined for each image separately. The standard deviation was calculated for the total brightness of each image:

(2) $$t_{i}=\bar{b_{i}}+5\sigma_{i}$$

where $\bar{b_{i}}$ denotes mean of brightness and $\sigma_{i}$ is a standard deviation of brightness of $i^{th}$ image. Finally, the threshold used for noise reduction has the form

(3) $$\begin{array}{c} threshold=\left\{\begin{array}{c} t_{i}\;\;\mathrm{for}\;\;t_{i}<100\\ 100\;\;\mathrm{for}\;\;t_{i}\geq 100 \end{array}\right. \end{array}$$

All pixels below the threshold are cut off. A set of images prepared in this way is subject to wavelet transform. More specifically, before feeding the images to the CNN, the Daubechies wavelet transformation was performed on them. Formally the original image signal f was transformed into four subimages according to the formula

(4) $$\begin{array}{c} f\to \left(\begin{array}{ccc} a & | & v\\ - & & -\\ h & | & d \end{array}\right), \end{array}$$

where subimage a denotes the average signal while h, v and d denote the horizontal, vertical and diagonal fluctuations, respectively [54]. All subimages have half the resolution of the original image.

**Figure 4:**
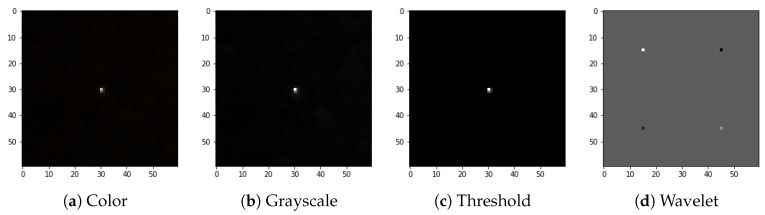
Preprocessing flow: (**a**) color input image, (**b**) grayscale accumulation, (**c**) adaptive thresholding, (**d**) wavelet transformation. Example of the spot-type image.

**Figure 5:**
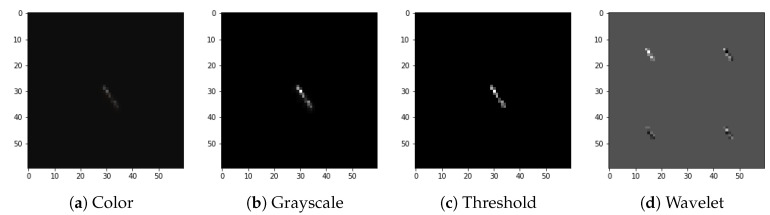
Preprocessing flow: (**a**) color input image, (**b**) grayscale accumulation, (**c**) adaptive thresholding, (**d**) wavelet transformation. Example of the track-type image.

**Figure 6:**
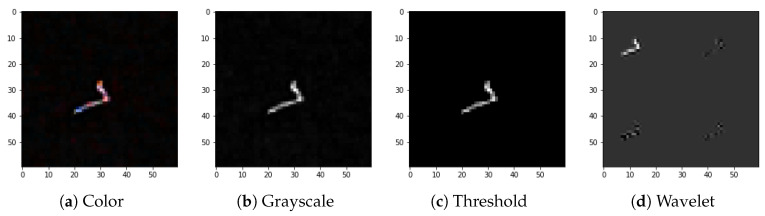
Preprocessing flow: (**a**) color input image, (**b**) grayscale accumulation, (**c**) adaptive thresholding, (**d**) wavelet transformation. Example of the worm-type image.

**Figure 7:**
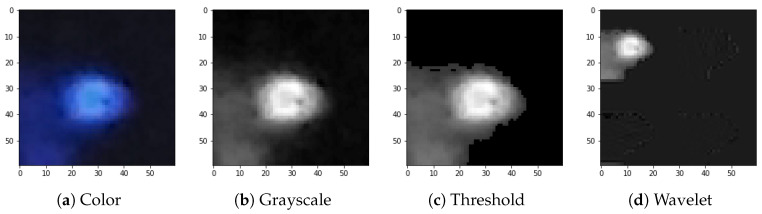
Preprocessing flow: (**a**) color input image, (**b**) grayscale accumulation, (**c**) adaptive thresholding, (**d**) wavelet transformation. Example of the artefact image.

The full preprocessing flow for exemplary images selected from the dataset is presented in Figure 4, Figure 5, Figure 6 and Figure 7.

### 2.4. Baseline Triggers

As already mentioned, the main rationale behind proposing the CNN-based trigger for CR detection in the CMOS cameras is the potential to easily extend this solution to any number of classes without essential changes in the network architecture, thus providing the consistence in signal processing. Still, it is instructive to compare the CNN-based trigger with a baseline classifiers which capture just the main differences between images attributable to signals and artefacts. There are indeed two qualitative features which enable the separation of signal and artefact images. These are the integrated luminosity (artefacts are generally brighter) and the number active pixels (in artefact images usually more pixels are lit). For the purposes of baseline triggers, both quantities, denoted *l* and np, respectively, take into account only the pixels above the threshold defined by Equation (3). Then, we determine the minimum integrated luminosity $l_{min}^{art}$ and minimum number of active pixels for images labelled as artefacts $np_{min}^{art}$ and maximal integrated luminosity $l_{max}^{sig}$ and maximal number of active pixels $np_{max}^{sig}$ for images labelled as signals. Given these quantities, the parameters determining the decision boundary are defined as $np_{b}=\left(np_{min}^{art}+np_{max}^{sig}\right)/2$ and $l_{b}=\left(l_{min}^{art}+l_{max}^{sig}\right)/2$. The decision boundary itself is thus defined as the quarter ellipse

(5) $${\left(\frac{np}{np_{b}}\right)}^{2}+{\left(\frac{l}{l_{b}}\right)}^{2}=1,\;\mathrm{with}\;np,l>0.$$

**Figure 8:**
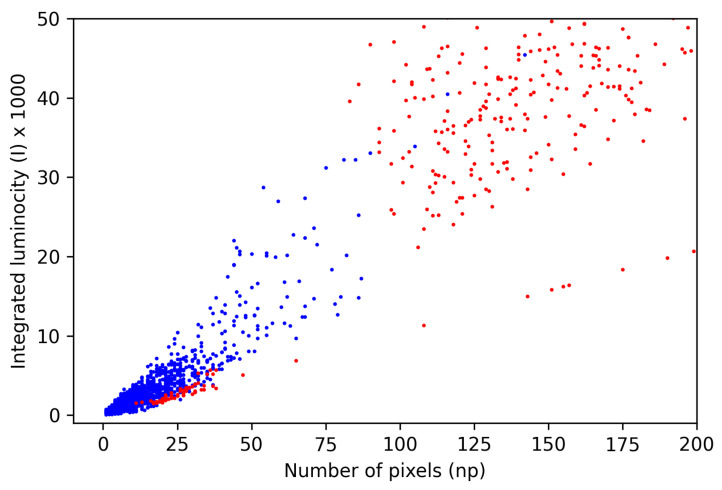
Structural distribution of samples belonging to each class in the feature space (partially scaled-up key area). Points assigned to signals and artefacts are marked blue and red, respectively.

All examples falling inside the quarter ellipse are classified as signals and those outside of it, as artefacts. The distribution of the signal and artefact labelled examples around the decision boundary is shown in Figure 8.

It is visible that the vast majority of signals lies within the decision boundary. However, still, there is some artefact admixture in this region. One can think about defining the decision boundary in a more elaborate way than that defined by Equation (5). To this end we tested the refined baseline triggers in the form of the kNN and Random Forest classifiers working in the same feature space as the base trigger. The performances of base trigger and its refined versions are summarised in Table 3. One sees that the baseline triggers perform surprisingly well, with the average signal and artefact recognition accuracy at the level of 96–97%. One also observes that the accuracy of the artefact recognition is about 4% worse across all baseline triggers. This difference can be attributed to the fraction of artefacts lying within decision boundary. This fraction could not be isolated out even with refined kNN and RF refined baseline triggers.

Note, however, that the overall high performance of the baseline triggers is reached at the cost of complete lack of generalisability, i.e., inability to work with increased number of classes. This is because the signals consisting of, e.g., straight lines (called tracks) and those consisting of curvy lines (called worms) and having the same number of active pixels, are entirely indistinguishable in this feature space.

## 3. Experimental Results

In this section, we discuss various preprocessing and training strategies which are aimed at the CNN-based trigger to follow the human annotators signal/artefact assignment as closely as possible. All computations have been performed on the Google Colaboratory platform using TensorFlow [55] and Keras libraries [56].

### 3.1. Training on Raw Data

**Figure 9:**
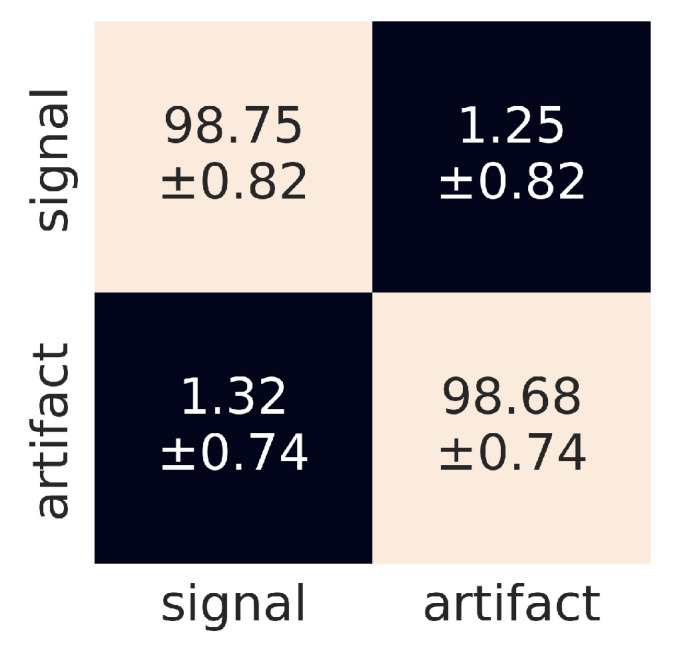
Confusion matrix for the raw data. The horizontal and vertical dimensions refer to predicted and judged labels, respectively.

In our base model, the raw un-preprocessed data were fed to the CNN. The objective of the base model was to evaluate and fine-tune the CNN architecture and to test the model’s vulnerability to the noise present in the original data. As shown in Table 4, the base model performs remarkably well in identification of both signals and artefacts, exceeding the accuracy of 98% in both cases. The corresponding confusion matrix is shown in Figure 9 and indicates that the distribution of the misclassified images is rather uniform.

**Figure 10:**
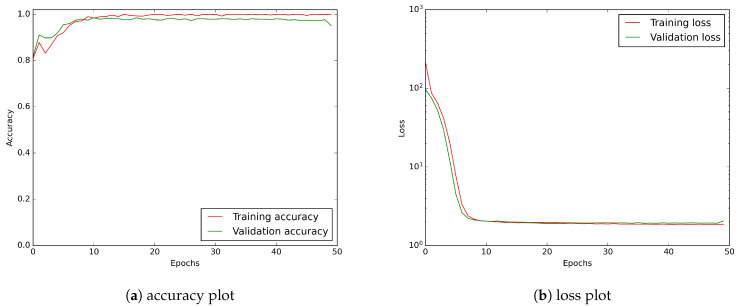
CNN model learning history showing accuracy and loss curves. A logarithmic scale has been applied to the loss plot to keep a better visibility.

In Figure 10, we have shown the accuracy and the loss function values vs. the number of epochs. Both of them show that the learning process saturates at about the 20th epoch. Thus, the 50 epoch training we use is more than satisfactory.

### 3.2. Training on Wavelet Transformed Data

In this section, we present the results obtained using the method discussed in Section 2. To select the optimal form of the input to the CNN, apart from the adaptive thresholding discussed in Section 2.3, we tested several types and combinations of Daubechies wavelet transforms available in Mahotas library [57]. In Table 5, we show the recognition accuracy rates for the input signals in the form of a single wavelet (1-dimensional input tensor). All results have been evaluated using the repeated 5-fold cross-validation. Apparently, the application of any type of the wavelet from the set (D2, D4, ..., D20) results in a recognition rate, for both signals and artefacts, equal to 98% within two standard deviations.

In Table 6, we show the accuracy results obtained with another approach, where the CNN was fed with wavelet tensors of varying depths in the range from D2:D4 (2 wavelets) to D2:D20 (10 wavelets). Again, very stable accuracy at the level of 98% was found across various wavelet sequences.

We attribute this accuracy stability to the fact that, even though both signals and artefacts are very diverse within their respective classes, there are clear morphological distinctions, e.g., in terms of the number of active pixels, between signals and artefacts. This can be observed by comparison of Figure 4, Figure 5, Figure 6 and Figure 7.

**Figure 11:**
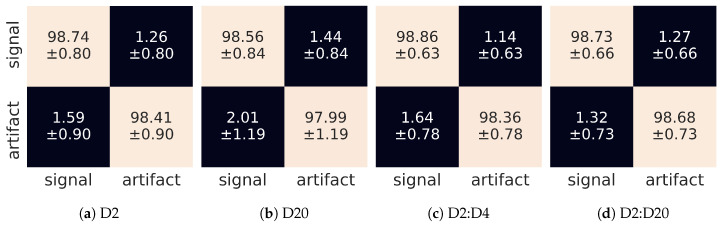
Confusion matrices for different input tensors: (**a**) single D2 wavelet, (**b**) single D20 wavelet, (**c**) composition of [D2,D4] wavelets, (**d**) composition of [D2,D4,D6,...,D20] wavelets. The horizontal and vertical dimensions refer to predicted and judged labels, respectively.

Finally, in Figure 11 we show confusion matrices, for both single wavelet and wavelet sequence versions of the experiment. Again, we see that (within one standard deviation) the misclassification rate is the same for signals and artefacts and is not worse than 2%.

**Figure 12:**
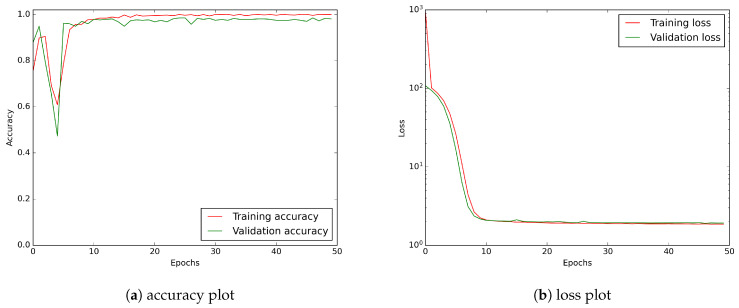
The exemplary of CNN model learning history for input tensor consisting of D2:D20 wavelets: (**a**) accuracy plot, (**b**) loss curve. A logarithmic scale has been applied to the loss plot to keep a better visibility.

As shown in Figure 12, the CNN learning curves for the wavelet transformed input stabilise around the 20th epoch.

### 3.3. Combined Approach

**Figure 13:**
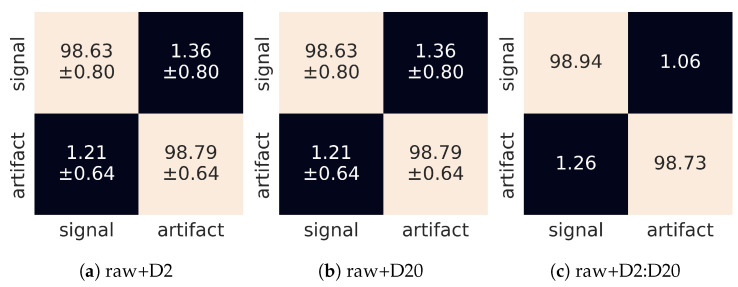
Confusion matrices for different dimensions of the input tensors: (**a**) composition of the raw image and single D2 wavelet, (**b**) composition of the raw image and single D20 wavelet, (**c**) composition of the raw image and collection of [D2, D4, ..., D20] wavelets. The horizontal and vertical dimensions refer to predicted and judged labels, respectively.

Finally, we explored the possibility to feed the CNN with both the raw data as well as the thresholded and then wavelet transformed data. This way, the model was exposed to effective feature extraction (by the wavelet transform), while retaining the information of the substantial noise component. Again, as can be observed in Table 7, the recognition rate exceeds 98% but compared to the training on raw data or wavelet transformed data separately, we do not observe substantial gain in combining the two approaches. The corresponding confusion matrices are shown in Figure 13.

**Figure 14:**
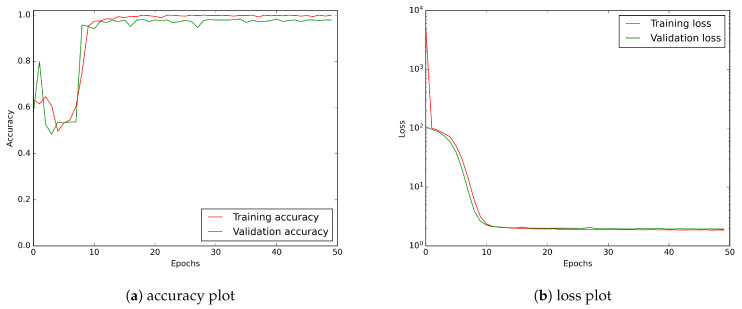
CNN model learning history for an input tensor consisting of raw data merged with D2:20 wavelets: (**a**) accuracy plot, (**b**) loss curve. A logarithmic scale has been applied to the loss plot to keep a better visibility.

Figure 14 indicates that similarly as in previous two cases the CNN learning curves for the combined input stabilise around 20th epoch.

### 3.4. Discussion of Experimental Results

The CNN classifier variants introduced in Section 3.1, Section 3.2 and Section 3.3 retain the same architecture but differ in the type and size of the input data. Despite this, their performance is comparable to within one standard deviation and achieves an accuracy close to 99%. To ascertain robustness and stability, verification of the obtained accuracy was performed using the k-fold cross-validation technique. The summary results of the computational experiments in this regard are given in Table 4, Table 5, Table 6 and Table 7. As the performance of the different variants of the classified do not differ significantly, in practical applications the model with lower time complexity should be favoured. The performance estimation of the different variants of the proposed classifier in this regard is presented in Table 8. These values indicate that it is worth using models requiring input data of the smallest possible size, i.e., raw images or single wavelets. This may be important in applications that require running the model directly on mobile devices (smartphones).

The learning curves shown in Figure 10, Figure 12 and Figure 14 exhibit some perturbations over the first few epochs. This is particularly strongly visible for models using tensor inputs containing wavelets. This phenomenon is probably due to rapid changes in model parameters during the initial learning phase. This is turn may be a consequence of the relatively small depth of the network and the small spatial size of the input images.

### 3.5. Demonstration of Models Performance

**Figure 15:**
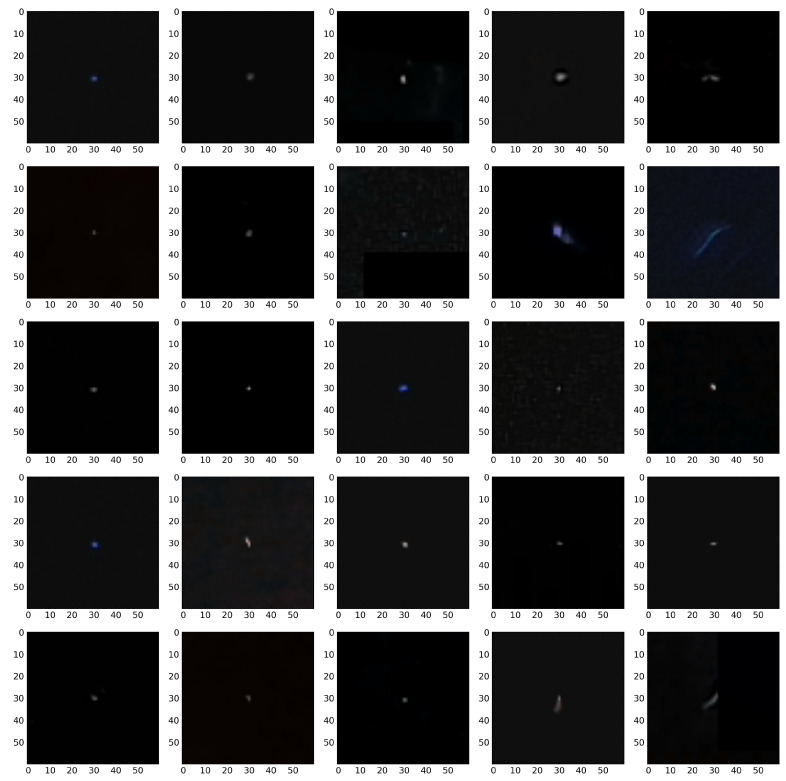
Classification on the selected part of the original CREDO set demonstrating signal class. An example of the performance of a classifier based on a dimension 2 input tensor composed of the greyscale image and a D2 wavelet.

**Figure 16:**
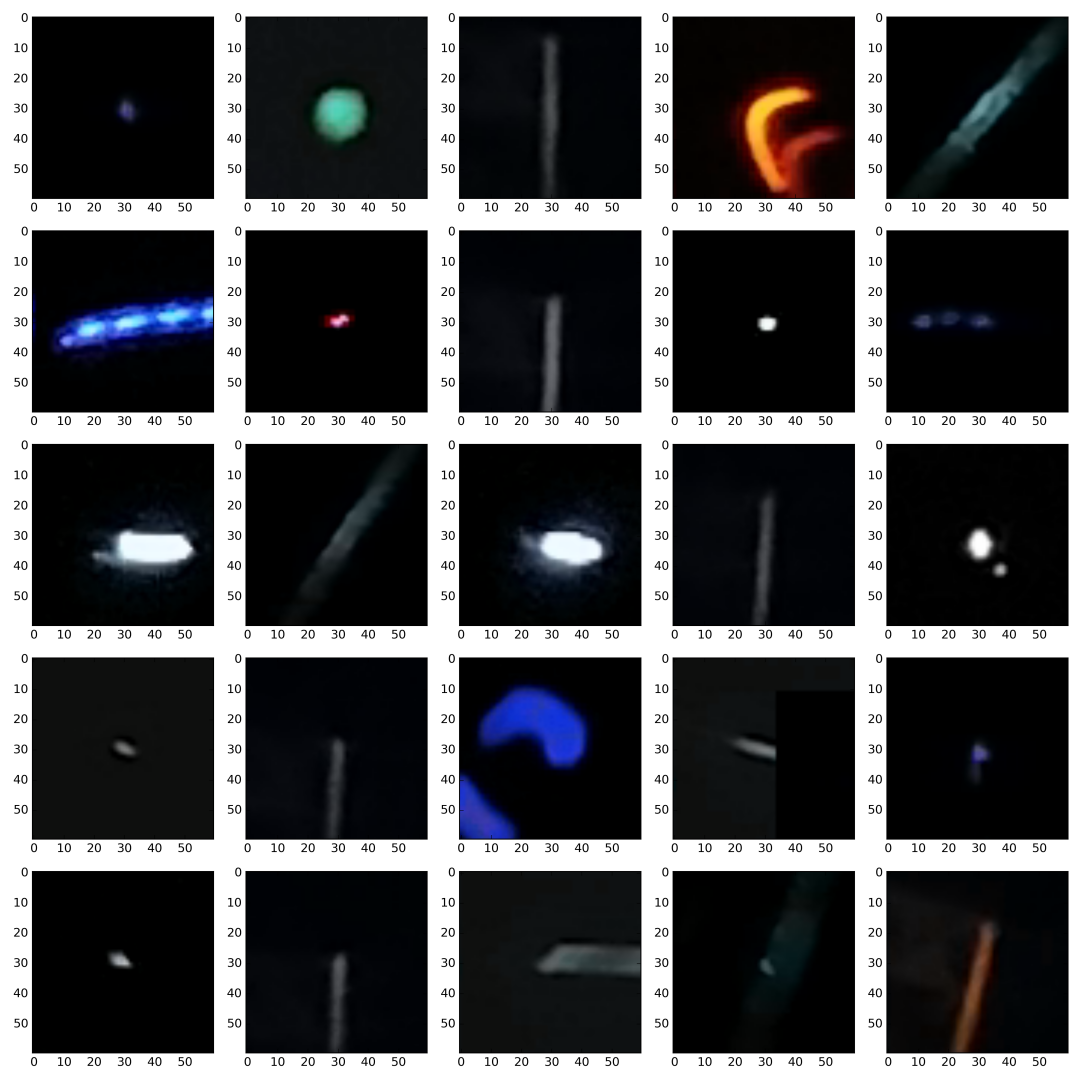
Classification on the selected part of original CREDO set demonstrating the artefact class. An example of the performance of a classifier based on a dimension 2 input tensor composed of the greyscale image and a D2 wavelet.

We want to stress one more time that at the present stage of investigation the only meaningful question one may ask in not how accurate the classification is but rather how accurately the trigger mimics the human annotators and how consistent it is in triggering. Figure 15 and Figure 16 show the random specimens of 25 images classified as signals and artefacts. Both figures show, albeit qualitatively, that the trigger is rather consistent. Providing more quantitative support of trigger accuracy requires larger set of annotated images. An alternative approach would be to cross check the CNN based trigger with an alternative trigger. We are currently performing such a study.

## 4. Discussion of Alternative Architectures

The main motivation behind the trigger architecture discussed in the preceding sections was to create a solution which, on the one hand, will be able to encompass the great variety of signal and artefact morphologies and, on the other hand, will easily generalise (without a change in network structure) to several signal classes. The canonical classes of signals observed in CCDs have been defined almost 20 years ago as spots, tracks and worms [58]. However, later CMOS-based observations, also by CREDO collaboration, suggested the emergence of multi-track signals, so the classifying network must be big enough to be able to accommodate such extended classification. Furthermore, given the current CREDO dataset size of several millions of images and its designed increase by two orders of magnitude, we have adopted preprocessing operations that are as simple and time efficient as possible. Therefore, having performed the wavelet transform, we refrained from further image segmentation but rather utilised the CNN’s capability to simultaneously process several sectors of each image, and then fed the four sub-images resulting from Equation (4) as a single image.

Now, it is tempting to check how these two assumptions (flat input and big network) impacted the overall classifier’s performance. To this end we performed two exploratory studies, discussed in the following two subsections, where we analysed an alternative input organisation, and secondly analysed the performance of the CNN with the number of input parameters reduced by an order of magnitude.

### 4.1. Alternative Input Organisation

The wavelet transform computed for a single image generates four components (**a**, **v**, **h**, **d**), with each component being half the size of the original input image. In the basic solution, these four components are spatially folded into a single image whose dimensions add up to the original image. This technique allows such wavelet subimages to be combined together with the original image into a single coherent tensor without scaling. In this section, we discuss another approach to constructing the input data tensor by treating individual wavelet components as separate layers. The formal definition of this way of constructing the wavelet representation as a multidimensional tensor is described by Equation (6).

(6) $$\begin{array}{c} f\to \left[a|v|h|d\right], \end{array}$$

where subimage a denotes the average signal while h, v and d denote the horizontal, vertical and diagonal fluctuations, respectively [54].

As a result, the wavelet representation generated for a single image takes the form of a tensor with dimensions half the size of the original image (30 × 30 px) and a depth of 4 layers. As the base model uses a set of transforms chosen in such a way that it can efficiently process input data with a resolution of 60 × 60 px, it is necessary to scale the twice smaller wavelet representation to this size. Without this rescaling, it would also not be possible to assemble the input data tensor containing the wavelets and raw image. The rescaling is done by interpolating the individual wavelet images to a higher resolution. This keeps the size of input tensors unchanged and allows a direct comparison of the obtained classification results, as the model architecture and the input data size are preserved.

In order to evaluate whether this arrangement of wavelet components noticeably affects the classification results, a corresponding experiment was conducted. Input data tensors constructed according to the newly proposed scheme, i.e., sequential arrangement of wavelet components, were loaded into the base classifier discussed in Section 2.2. These tensors were also supplemented with an additional layer in the form of a greyscale image to enrich information about the global luminance distribution. The results obtained are shown in Table 9. They are comparable in terms of standard deviation accuracy with the previously obtained results for the base model. Thus, there is no noticeable effect of the wavelet component setting on the model performance.

### 4.2. Application of Smaller Scale Model

Besides the input data format, the second extremely important aspect is the model architecture itself. The proposed base model by its scale far exceeds the size of the learning set. The model itself is not very deep (4 convolutional layers), but it is quite broad in the sense that it uses a significant number of filters in each layer. This results in a model size of about 1 million parameters requiring learning and tuning. Compared to the size of the training set, which contains about 2000 elements, there may be reasonable doubt as to whether such a difference in scale compromises the ability to effectively learn such a model from the available data.

**Figure 17:**
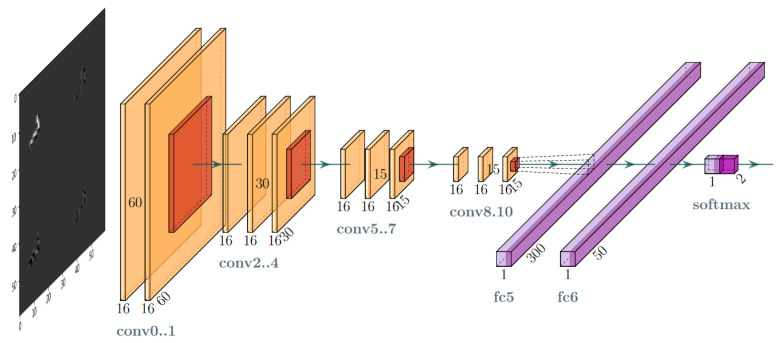
Layer-oriented signal flow in the convolutional network (CNN) artefact filtration model of a smaller scale.

To verify this issue, a dedicated smaller scale model was developed for comparison purposes. During the development of the small scale model, it became apparent that it needed to be much deeper than the base model in order to learn effectively from the available data. The result of many design trials and experiments is the architecture of the convolutional network shown in Table 10 and in illustrative form in Figure 17. The final model has about 100,000 parameters requiring learning and tuning, so it is an order of magnitude smaller than the baseline model. Consequently, this model should be less susceptible to overfitting than the baseline model.

This small-scale model was then trained on a standard dataset to compare the performance and statistical parameters of the classification process with that of the base model. To this end, both of the wavelet tensor ordering techniques discussed previously were used, i.e., combining subimages of a single layer and combining subimages into a sequence of layers. In addition, in both cases the tensor was extended with a layer containing the greyscale source image. Table 11 and Table 12 present the obtained wavelet tensor classification results ordered according to the schemes described by Formulas (4) and (6), respectively.

Comparing the classification results obtained with the base model and the small-scale model, it cannot be concluded that they differ significantly from each other. With respect to the determined standard deviations, the analysed models show comparable performance. Thus, in our opinion, it can be concluded that the use of the base model is justified even in the case of a large scale difference with respect to the power of the available training set.

A baseline model using an architecture with more learning parameters certainly has much more potential in terms of discriminative ability and intrinsic feature representation capacity. In this sense, it may be promising to use it as a prototype solution for more demanding applications, such as multi-class classification of signals distinguishing their different morphologies.

### 4.3. Summary of Alternative Architectures

To summarise our exploratory studies towards modified shape of the input tensor and the decreased number of CNN’s units, we conclude neither replacing the single wavelet image with a tensor dimension equal to four nor the decrease of the number of neurons by one order of magnitude do not change significantly the classifier’s performance. Thus, given the original requirements of fast image preprocessing and the network’s ability to accommodate multi-class classification, we conclude that the original trigger setup is the right base for larger dataset trigger.

## 5. Summary and Outlook

We described an application of a Convolutional Neural Network to filter artefacts in the cosmic ray detection experiments performed on mobile phones. Generally, such experiments are aimed at broader scientifically oriented audience, in the framework of the so called Citizen Science philosophy. A gamification (e.g., Particle Hunters’ Competitions) is an efficient method to sustain the participants’ engagement, necessary for such projects to be scientifically productive. However, the gamification is accompanied by the surge of fake signals related either to the hardware malfunction or participants’ cheating. Our method uses a subset of CREDO images labelled by judges as either “signal” or “artefact”. We started from considering a baseline trigger whose training consisted on constructing the decision boundary in two-dimensional feature space defined by integrated luminosities and the number of active pixels. On average the baseline trigger and its refined versions based on kNN and RF classifiers performed just 2% worse than the CNN trigger. Their artefact recognition rate was, however, 4% worse than that of the CNN trigger. Then, we have studied three versions of the experiment setup and two architectures of CNN models. In the basic version, the raw CR images were fed to the CNN. In the refined version of our solution, the images were adaptively thresholded and then subject to wavelet transforms. The motivation of the wavelet transform was its ability to amplify distinctive signal features, like the shape of object borders or its fragmentation. Such input was then fed to the CNN. Finally, we have studied the impact of simultaneous feeding of raw and wavelet transformed data but found no significant improvement of the recognition rate. The overall accuracy of three discussed approaches reached the level of 98–99% for both signal and artefacts. With such accuracies the adverse effects of gamification can be effectively neutralised. Given the similar performance of all three preprocessing methods the practical application of the method is determined by time efficiency which favours the raw RGB based CNN classification.

In general, the classifiers investigated are limited to some extent by the accuracy of the annotators in recognising whether a hit is a signal or artefact. As shown in the paper, CNN triggers were found to be significantly more consistent than annotators (smaller standard deviation). The natural extension of the presented methods is to increase the number of signal classes so that various types of particle tracks can be identified. This research is currently under way.

## Figures and Tables

**Table 1:** All participants vs. competition participants. Data collected from 1 October 2019 to 30 January 2020).

	All	Competitors	(% of All)
Users	1533	717	(47%)
Teams	310	62	(21%)
Devices	1756	836	(48%)
Total time work [Hours]	459,000	170,000	(37%)
Candidates of detections	1,207,000	812,000	(67%)
Good Detections	558,000	242,000	(46%)
% of good candidates	46%	30%	

**Table 2:** Layer-by-layer summary of the proposed CNN model. Each layer name is given followed by the number of feature maps (convolutional layers) or neurons (dense layers), the size of the convolutional filter or pooling region, the activation function used and, last, the number of parameters to learn.

Layer Type	Features	Size	Activation	Params
Convolution 2D	32	5 × 5	ReLU	832
Max Pooling	-	2 × 2	-	-
Convolution 2D	64	5 × 5	ReLU	51,264
Max Pooling	-	2 × 2	-	-
Convolution 2D	128	5 × 5	ReLU	204,928
Max Pooling	-	2 × 2	-	-
Convolution 2D	128	5 × 5	ReLU	409,728
Max Pooling	-	2 × 2	-	-
Flatten	1152	-	-	-
Dense	300	-	ReLU	345,900
Dense	50	-	ReLU	15,050
Dense	2	-	Softmax	102
Total params: 1,027,804				
Trainable params: 1,027,804				
Non-trainable params: 0				

**Table 3:** Performance of the base model applied to the input data. Three variants of classifiers using manually selected two features were analysed: a simple heuristic model based on decision rules, a kNN type classifier (k = 7, metric = L2) and a classifier based on boosted decision trees called random forests (number_estimators = 100, depth_trees = 2). Results have been estimated using repeated k-fold validation (5 rounds with 5 folds each).

Type	Overall Acc ± Std Dev	Signal Acc ± Std Dev	Artefact Acc ± Std Dev
Baseline trigger	96.87 ± 0.66	98.65 ± 1.10	94.92 ± 1.14
Nearest Neighbor	96.40 ± 0.73	98.57 ± 0.60	94.01 ± 1.27
Random Forest	97.07 ± 0.77	99.17 ± 0.73	94.76 ± 1.39

**Table 4:** CNN model performance for raw data set. Results have been estimated using repeated k-fold validation (5 rounds with 5 folds each).

Tensor Depth	Overall Acc ± Std Dev	Signal Acc ± Std Dev	Artefact Acc ± Std Dev
3	98.71± 0.50	98.75 ± 0.82	98.68 ± 0.74

**Table 5:** CNN model performance for various single wavelet transformations applied to input data. Results have been estimated using repeated k-fold validation (5 rounds with 5 folds each).

Wavelet	Tensor	Overall Acc	Signal Acc	Artefact Acc
Number	Depth	± Std Dev	± Std Dev	± Std Dev
D2	1	98.56 ± 0.62	98.74 ± 0.80	98.41 ± 0.90
D4	1	98.54 ± 0.48	98.51 ± 0.86	98.45 ± 0.99
D6	1	98.48 ± 0.46	98.51 ± 0.86	98.45 ± 0.99
D8	1	98.48 ± 0.46	98.51 ± 0.86	98.45 ± 0.99
D10	1	98.62 ± 0.49	99.01 ± 0.66	98.22 ± 0.78
D12	1	98.63 ± 0.49	98.88 ± 0.69	98.34 ± 0.73
D14	1	98.53 ± 0.56	98.60 ± 0.91	98.45 ± 0.83
D16	1	98.44 ± 0.56	98.60 ± 0.91	98.45 ± 0.83
D18	1	98.38 ± 0.40	98.49 ± 0.64	98.25 ± 0.94
D20	1	98.28 ± 0.57	98.55 ± 0.83	97.99 ± 1.19

**Table 6:** CNN model performance for various wavelet transformations applied to input data (deep tensors). Results have been estimated using repeated k-fold validation (5 rounds with 5 folds each).

Wavelets	Tensor	Overall Acc	Signal Acc	Artefact Acc
Sequence	Depth	± Std Dev	± Std Dev	± Std Dev
D2:D4	2	98.62 ± 0.41	98.86 ± 0.63	98.36 ± 1.64
D2:D6	3	98.65 ± 0.54	98.62 ± 0.77	98.68 ± 0.85
D2:D8	4	98.65 ± 0.54	98.62 ± 0.62	98.68 ± 0.92
D2:D10	5	98.71 ± 0.49	98.83 ± 0.74	98.57 ± 0.65
D2:D12	6	98.60 ± 0.39	98.75 ± 0.85	98.43 ± 0.92
D2:D14	7	98.67 ± 0.40	99.01 ± 0.67	98.29 ± 0.76
D2:D16	8	98.64 ± 0.50	98.83 ± 0.68	98.43 ± 0.88
D2:D18	9	98.73 ± 0.47	98.86 ± 0.83	98.59 ± 0.83
D2:D20	10	98.71 ± 0.47	98.73 ± 0.66	98.68 ± 0.73

**Table 7:** CNN model performance for raw data combined with wavelet set. Results have been estimated using repeated k-fold validation (5 rounds with 5 folds each).

Data	Tensor	Overall Acc	Signal Acc	Artefact Acc
Set	Depth	± Std Dev	± Std Dev	± Std Dev
raw + D2	2	98.93 ± 0.39	98.99 ± 0.63	98.86 ± 0.67
raw + D20	2	98.71 ± 0.43	98.64 ± 0.80	98.79 ± 0.64
raw + D2:D20	11	98.84 ± 0.55	98.94 ± 0.71	98.73 ± 0.95

**Table 8:** CNN model timing for different type of input data, where raw denotes the original RGB image and Dn means Daubechies wavelet with length of *n*. To obtain statistically reliable results, classification time was measured for $10^{5}$ samples and then averaged.

Data Set	Tensor Depth	Learning Time [s]	Prediction Time [μs]
		for 50 Epochs	for Single Image
raw	3	27	100
D2	1	29	110
raw + D2	4	29	140
raw + D2:D10	8	33	180
raw + D2:D20	13	39	260

**Table 9:** Base CNN model performance for various single wavelet transformations applied to input data. Input tensor composed of a greyscale image and wavelet image components (**a**, **v**, **h**, **d**) set in a sequence which gives a tensor depth of 1 + 4. Results have been estimated using repeated k-fold validation (5 rounds with 5 folds each).

Wavelet	Tensor	Overall Acc	Signal Acc	Artefact Acc
Number	Depth	± Std Dev	± Std Dev	± Std Dev
raw + D2	5	98.11 ± 0.61	97.92 ± 0.91	98.32 ± 1.09
raw + D4	5	98.10 ± 0.49	97.71 ± 0.86	98.54 ± 0.66
raw + D6	5	97.92 ± 0.69	97.84 ± 0.96	97.90 ± 1.17
raw + D8	5	97.99 ± 0.56	97.95 ± 1.01	98.02 ± 1.11
raw + D10	5	98.03 ± 0.55	98.02 ± 0.96	98.04 ± 0.91
raw + D12	5	98.10 ± 0.65	97.79 ± 1.06	98.43 ± 0.89
raw + D14	5	98.10 ± 0.57	98.26 ± 0.85	97.90 ± 0.81
raw + D16	5	98.13 ± 0.52	98.18 ± 0.58	98.07 ± 0.97
raw + D18	5	97.95 ± 0.56	97.97 ± 0.78	97.93 ± 1.23
raw + D20	5	98.04 ± 0.46	98.07 ± 0.70	98.00 ± 0.73

**Table 10:** Layer-by-layer summary of the proposed smaller-scale CNN model. Each layer name is given followed by the number of feature maps (convolutional layers) or neurons (dense layers), the size of the convolutional filter or pooling region, the activation function used and, last, the number of parameters to learn.

Layer Type	Features	Size	Activation	Params
Convolution 2D	16	3 × 3	ReLU	736
Convolution 2D	16	5 × 5	ReLU	6416
Max Pooling	-	2 × 2	-	-
Convolution 2D	16	3 × 3	ReLU	2320
Convolution 2D	16	3 × 3	ReLU	2320
Convolution 2D	16	5 × 5	ReLU	6416
Max Pooling	-	2 ×2	-	-
Convolution 2D	16	3 × 3	ReLU	2320
Convolution 2D	16	3 × 3	ReLU	2320
Convolution 2D	16	5 × 5	ReLU	6416
Max Pooling	-	2 × 2	-	-
Convolution 2D	16	3 × 3	ReLU	2320
Convolution 2D	16	3 × 3	ReLU	2320
Convolution 2D	16	5 × 5	ReLU	6416
Max Pooling	-	2 × 2	-	-
Flatten	144	-	-	-
Dense	300	-	ReLU	43,500
Dense	50	-	ReLU	15,050
Dense	2	-	Softmax	102
Total params: 98,972				
Trainable params: 98,972				
Non-trainable params: 0				

**Table 11:** Smaller-scale CNN model performance for various single wavelet transformations applied to input data. Results have been estimated using repeated k-fold validation (5 rounds with 5 folds each).

Wavelet	Tensor	Overall Acc	Signal Acc	Artefact Acc
Number	Depth	± Std Dev	± Std Dev	± Std Dev
raw + D2	2	98.33 ± 0.58	98.49 ± 0.67	98.15 ± 1.14
raw + D4	2	98.12 ± 0.88	98.33 ± 1.02	97.90 ± 1.22
raw + D6	2	98.26 ± 0.48	98.41 ± 0.69	98.09 ± 0.89
raw + D8	2	98.38 ± 0.60	98.46 ± 1.08	98.29 ± 0.80
raw + D10	2	98.27 ± 0.48	98.59 ± 0.65	97.91 ± 0.84
raw + D12	2	98.30 ± 0.61	98.52 ± 0.82	98.06 ± 1.06
raw + D14	2	98.13 ± 0.55	98.28 ± 0.71	97.97 ± 0.96
raw + D16	2	98.18 ± 0.53	98.36 ± 0.86	97.99 ± 0.89
raw + D18	2	98.14 ± 0.57	98.10 ± 0.98	98.18 ± 0.85
raw + D20	2	98.17 ± 0.54	98.30 ± 0.83	98.04 ± 0.73

**Table 12:** Smaller-scale CNN model performance for various single wavelet transformations applied to input data. Input tensor composed of a greyscale image and wavelet image components (**a**, **v**, **h**, **d**) set in a sequence which gives a tensor depth of 1 + 4. Results have been estimated using repeated k-fold validation (5 rounds with 5 folds each).

Wavelet	Tensor	Overall Acc	Signal Acc	Artefact Acc
Number	Depth	± Std Dev	± Std Dev	± Std Dev
raw + D2	5	98.16 ± 0.54	98.10 ± 0.88	98.22 ± 0.89
raw + D4	5	98.10 ± 0.48	98.02 ± 0.82	98.20 ± 0.84
raw + D6	5	97.99 ± 0.54	97.99 ± 0.88	98.00 ± 1.10
raw + D8	5	98.02 ± 0.35	97.89 ± 0.93	98.18 ± 0.94
raw + D10	5	97.87 ± 0.64	97.74 ± 0.97	98.00 ± 0.92
raw + D12	5	97.91 ± 0.69	97.82 ± 1.05	98.00 ± 0.86
raw + D14	5	97.82 ± 0.61	97.94 ± 1.08	97.68 ± 1.11
raw + D16	5	97.99 ± 0.58	98.04 ± 0.99	97.95 ± 0.96
raw + D18	5	97.99 ± 0.42	97.68 ± 1.12	97.91 ± 1.10
raw + D20	5	97.86 ± 0.59	97.79 ± 1.03	97.93 ± 0.86

## Data Availability

The training set as well as the source code used in this analysis are available at https://github.com/credo-ml/cnn-offline-trigger, accessed on 9 July 2021.

## Abbreviations

The following abbreviations are used in this manuscript:

CREDO	Cosmic Ray Extremely Distributed Observatory
CNN	Convolutional Neural Network
RNN	Recurrent Neural Networks
GNN	Graph Neural Networks
ReLU	Rectified Linear Unit
ANN	Artificial Neural Network
SVM	Support Vector Machine
RF	Random Forest
DECO	Distributed Electronic Cosmic-ray Observatory
CCD	Charge Coupled Device
CMOS	Complementary Metal-Oxide-Semiconductor
CRAYFIS	Cosmic RAYs Found In Smartphones

## References

[1] Homola P., Beznosko D., Bhatta G., Bibrzycki Ł, Borczyńska M., Bratek Ł, Budnev N., Burakowski D., Alvarez-Castillo D.E., Almeida Cheminant K. (2020). Cosmic-Ray Extremely Distributed Observatory. Symmetry.

[2] Unger M., Farrar G. (2015). (In) Feasability of Studying Ultra-High-Energy Cosmic Rays with Smartphones. arXiv.

[3] Kumar R. Tracking Cosmic Rays by CRAYFIS (Cosmic Rays Found in Smartphones) Global Detector. Proceedings of the 34th International Cosmic Ray Conference (ICRC2015).

[4] Borisyak M., Usvyatsov M., Mulhearn M., Shimmin C., Ustyuzhanin A. (2017). Muon trigger for mobile phones. J. Phys..

[5] Albin E., Whiteson D. (2021). Feasibility of Correlated Extensive Air Shower Detection with a Distributed Cosmic Ray Network. arXiv.

[6] Whiteson D., Mulhearn M., Shimmin C., Cranmer K., Brodie K., Burns D. (2016). Searching for ultra-high energy cosmic rays with smartphones. Astropart. Phys..

[7] Winter M., Bourbeau J., Bravo S., Campos F., Meehan M., Peacock J., Ruggles T., Schneider C., Simons A.L., Vandenbroucke J. (2019). Particle identification in camera image sensors using computer vision. Astropart. Phys..

[8] Vandenbroucke J., Bravo S., Karn P., Meehan M., Plewa M., Ruggles T., Schultz D., Peacock J., Simons A.L. (2015). Detecting particles with cell phones: The Distributed Electronic Cosmic-ray Observatory. arXiv.

[9] Vandenbroucke J., BenZvi S., Bravo S., Jensen K., Karn P., Meehan M., Peacock J., Plewa M., Ruggles T., Santander M. (2016). Measurement of cosmic-ray muons with the Distributed Electronic Cosmic-ray Observatory, a network of smartphones. J. Instrum..

[10] Meehan M., Bravo S., Campos F., Peacock J., Ruggles T., Schneider C., Simons A.L., Vandenbroucke J., Winter M. (2017). The particle detector in your pocket: The Distributed Electronic Cosmic-ray Observatory. arXiv.

[11] De Angelis A., Pimenta M. (2018). Introduction to Particle and Astroparticle Physics.

[12] Collaboration H., Abramowski A., Aharonian F. (2016). Acceleration of petaelectronvolt protons in the Galactic Centre. Nature.

[13] Webb G.M., Al-Nussirat S., Mostafavi P., Barghouty A.F., Li G., le Roux J.A., Zank G.P. (2019). Particle Acceleration by Cosmic Ray Viscosity in Radio-jet Shear Flows. Astrophys. J..

[14] Globus N., Blandford R.D. (2020). The Chiral Puzzle of Life. Astrophys. J..

[15] (2016). Catalogue of electron precipitation events as observed in the long-duration cosmic ray balloon experiment. J. Atmos. Sol. Terr. Phys..

[16] Chancellor J.C., Scott G.B.I., Sutton J.P. (2014). Space Radiation: The Number One Risk to Astronaut Health beyond Low Earth Orbit. Life.

[17] Mavromichalaki H., Papailiou M., Dimitrova S., Babayev E.S., Loucas P. (2012). Space weather hazards and their impact on human cardio-health state parameters on Earth. Nat. Hazards.

[18] The CREDO Collaboration (2021). CREDO Detector. https://github.com/credo-science/credo-detector-android.

[19] Bibrzycki Ł., Burakowski D., Homola P., Piekarczyk M., Nied´zwiecki M., Rzecki K., Stuglik S., Tursunov A., Hnatyk B., Castillo D.E.A. (2020). Towards A Global Cosmic Ray Sensor Network: CREDO Detector as the First Open-Source Mobile Application Enabling Detection of Penetrating Radiation. Symmetry.

[20] Particle Hunters—CREDO Competition. https://credo.science/particle_hunters/).

[21] Murphy K.P. (2012). Machine Learning: A Probabilistic Perspective.

[22] James G., Witten D., Hastie T., Tibshirani R. (2014). An Introduction to Statistical Learning: With Applications in R.

[23] Cortes C., Vapnik V. (1995). Support-vector networks. Mach. Learn..

[24] Ripley B.D. (2007). Pattern Recognition and Neural Networks.

[25] Biau G., Scornet E. (2016). A random forest guided tour. Test.

[26] Li Z., Yang W., Peng S., Liu F. (2020). A survey of convolutional neural networks: Analysis, applications, and prospects. arXiv.

[27] Ghatak A. (2019). Recurrent neural networks (RNN) or sequence models. Deep Learning with R.

[28] Wu Z., Pan S., Chen F., Long G., Zhang C., Philip S.Y. (2020). A comprehensive survey on graph neural networks. IEEE Trans. Neural Netw. Learn. Syst..

[29] Yaneva V., Eraslan S., Yesilada Y., Mitkov R. (2020). Detecting high-functioning autism in adults using eye tracking and machine learning. IEEE Trans. Neural Syst. Rehabil. Eng..

[30] Mekov E., Miravitlles M., Petkov R. (2020). Artificial intelligence and machine learning in respiratory medicine. Expert Rev. Respir. Med..

[31] Huang K., Bryant T., Schneider B. Identifying Collaborative Learning States Using Unsupervised Machine Learning on Eye-Tracking, Physiological and Motion Sensor Data. Proceedings of the 12th International Conference on Educational Data.

[32] Sharma K., Giannakos M., Dillenbourg P. (2020). Eye-tracking and artificial intelligence to enhance motivation and learning. Smart Learn. Environ..

[33] Tomczyk K., Piekarczyk M., Sieja M., Sokal G. (2021). Special functions for the extended calibration of charge-mode accelerometers. Precis. Eng..

[34] Tomczyk K., Piekarczyk M., Sokal G. (2019). Radial basis functions intended to determine the upper bound of absolute dynamic error at the output of voltage-mode accelerometers. Sensors.

[35] Wang L., Wang Y. Application of Machine Learning for Process Control in Semiconductor Manufacturing. Proceedings of the 2020 International Conference on Internet Computing for Science and Engineering.

[36] Bibi K., Naz S., Rehman A. (2020). Biometric signature authentication using machine learning techniques: Current trends, challenges and opportunities. Multimed. Tools Appl..

[37] Kim S.K., Yeun C.Y., Damiani E., Lo N.W. (2019). A machine learning framework for biometric authentication using electrocardiogram. IEEE Access.

[38] Wójcik K., Piekarczyk M. (2020). Machine Learning Methodology in a System Applying the Adaptive Strategy for Teaching Human Motions. Sensors.

[39] Fang B., Jia S., Guo D., Xu M., Wen S., Sun F. (2019). Survey of imitation learning for robotic manipulation. Int. J. Intell. Robot. Appl..

[40] Hachaj T., Piekarczyk M. (2019). Evaluation of pattern recognition methods for head gesture-based interface of a virtual reality helmet equipped with a single IMU sensor. Sensors.

[41] Nogales R., Benalcázar M.E. (2019). A survey on hand gesture recognition using machine learning and infrared information. International Conference on Applied Technologies.

[42] Krizhevsky A., Sutskever I., Hinton G.E. (2012). Imagenet classification with deep convolutional neural networks. Adv. Neural Inf. Process. Syst..

[43] He K., Zhang X., Ren S., Sun J. (2015). Deep Residual Learning for Image Recognition. arXiv.

[44] Chollet F. Xception: Deep learning with depthwise separable convolutions. Proceedings of the IEEE Conference on Computer Vision and Pattern Recognition.

[45] Huang G., Liu Z., Van Der Maaten L., Weinberger K.Q. Densely connected convolutional networks. Proceedings of the IEEE Conference on Computer Vision and Pattern Recognition.

[46] Simonyan K., Zisserman A. (2014). Very deep convolutional networks for large-scale image recognition. arXiv.

[47] Sandler M., Howard A., Zhu M., Zhmoginov A., Chen L.C. Mobilenetv2: Inverted residuals and linear bottlenecks. Proceedings of the IEEE Conference on Computer Vision and Pattern Recognition.

[48] Russakovsky O., Deng J., Su H., Krause J., Satheesh S., Ma S., Huang Z., Karpathy A., Khosla A., Bernstein M. (2015). Imagenet large scale visual recognition challenge. Int. J. Comput. Vis..

[49] Hachaj T., Bibrzycki Ł, Piekarczyk M. (2021). Recognition of Cosmic Ray Images Obtained from CMOS Sensors Used in Mobile Phones by Approximation of Uncertain Class Assignment with Deep Convolutional Neural Network. Sensors.

[50] Niedźwiecki M., Rzecki K., Marek M., Homola P., Smelcerz K., Castillo D.A., Smolek K., Hnatyk B., Zamora-Saa J., Mozgova A. (2019). Recognition and classification of the cosmic-ray events in images captured by CMOS/CCD cameras. arXiv.

[51] Niedźwiecki M. (2021). Manual Classification of CREDO Cosmic Ray Traces. https://credo.nkg-mn.com/.

[52] Hinton G., Srivastava N., Swersky K. (2012). Neural networks for machine learning lecture 6a overview of mini-batch gradient descent. Cited.

[53] Zou H., Hastie T. (2005). Regularization and variable selection via the elastic net. J. R. Stat. Soc. Ser. B.

[54] Walker J. (1999). A Primer on Wavelets and Their Scientific Applications.

[55] Abadi M., Agarwal A., Barham P., Brevdo E., Chen Z., Citro C., Corrado G.S., Davis A., Dean J., Devin M. (2015). TensorFlow: Large-Scale Machine Learning on Heterogeneous Systems. http://tensorflow.org.

[56] Chollet F. (2015). Keras. https://github.com/fchollet/keras.

[57] Coelho L. (2013). Mahotas: Open source software for scriptable computer vision. J. Open Res. Softw..

[58] Groom D. (2002). Cosmic rays and other nonsense in astronomical CCD imagers. Exp. Astron..

